# Leptin, ghrelin and calprotectin: inflammatory markers in childhood asthma?

**DOI:** 10.1186/2049-6958-8-62

**Published:** 2013-09-25

**Authors:** Nazan Cobanoglu, Nilufer Galip, Ceyhun Dalkan, Nerin Nadir Bahceciler

**Affiliations:** 1 Department of Pediatric Pulmonology, Near East University, Faculty of Medicine, Nicosia, North Cyprus; 2 Department of Pediatric Allergy and Immunology, Near East University, Faculty of Medicine, Nicosia, North Cyprus; 3 Department of Pediatrics, Near East University, Faculty of Medicine, Nicosia, North Cyprus

**Keywords:** Asthma, Calprotectin, Ghrelin, Inflammation, Leptin

## Abstract

**Background:**

Appetite-modulating hormones ghrelin and leptin might be relevant to asthma with their pro-inflammatory effects, and calprotectin has been recognized as a promising marker of inflammation. The purpose of this study was to explore whether asthma, atopy and lung functions has a relation with serum levels of leptin, ghrelin and calprotectin as inflammatory markers in children.

**Methods:**

A cross-sectional study was performed by searching the doctor diagnosed asthma through questionnaires filled in by parents who were phoned, and children were invited to supply fasting blood samples in order to measure serum levels of leptin, ghrelin and calprotectin, and to perform skin prick test and spirometry. Participants were divided into Group 1, children with previous diagnosis of asthma, and Group 2, children without previous diagnosis of asthma.

**Results:**

One thousand and two hundred questionnaires were distributed and 589 of them were returned filled in. Out of 74 children whose parents accepted to participate in the study, 23 were in Group 1 and 51 were in Group 2. There was no statistical difference in serum levels of leptin, ghrelin, calprotectin, forced expiratory volume in one second (FEV_1)_, forced vital capacity (FVC), peak expiratory flow (PEF) , forced expiratory flow between 25 and 75% of vital capacity (FEF_25-75_) values , and skin prick test results between the two groups (p values are 0.39, 0.72, 0.5, 0.17, 0.5, 0.27, 0.18, and 0.81 respectively).

**Conclusion:**

In this study the inflammation in asthmatic children could not be shown by using serum leptin, ghrelin and calprotectin levels and this is possibly due to the low number of children with ever asthma and equal skin prick test positivity in both groups. This study is the first study aimed to show the relation between serum calprotectin levels and inflammation in asthma. As this study was a cross-sectional study, further prospectively designed randomized controlled studies are necessary to show the association of these markers and inflammation in asthma.

## Background

Asthma is a chronic inflammatory condition of the airways associated with airway hyper-responsiveness that leads to recurrent episodic of airflow obstruction within the lungs. Although the cause of childhood asthma has not been definitely determined, current research includes a combination of environmental respiratory exposures, such as inhaled allergens, respiratory viral infections, air pollutants, and genetic vulnerabilities.

Both the human and murine studies showed that the pro-inflammatory effects of recently discovered appetite-modulating hormones ghrelin and leptin might be relevant to asthma [1-3]. Both hormones have some immunomodulatory effects and counteract each other with regard to the production of pro-inflammatory cytokines such as TNF-α and IL-1β on human lymphocytes *in vitro*[4]. On the other hand, inflammation decreases serum ghrelin levels [5] and increases serum leptin levels [6]. Calprotectin has been recognized as a promising marker of inflammation [7,8]. The serum level of calprotectin may be a very sensitive non-specific inflammatory marker in various clinical settings and has been proposed for the diagnosis of many inflammatory conditions [9]. To date it has not been searched whether serum calprotectin levels with leptin and ghrelin levels could be used to detect the inflammation related to childhood asthma. The purpose of this study therefore was to explore whether asthma, atopy and lung functions have a relation with serum levels of leptin, ghrelin and calprotectin as inflammatory markers in children.

## Methods

A cross-sectional study was performed from May 1 to September 1, 2011, in summer time, among the first to fifth grade students of a randomly selected primary school in Nicosia. The investigation was coordinated by Near East University Faculty of Medicine Department of Paediatrics and approved by the Ethics Committee of this faculty. The International Study of Asthma and Allergies in Childhood (ISAAC) questionnaire, translated into Turkish previously [10], was distributed to all students to be filled in by their parents and returned to school. Then all responders were phoned, given information about the study and their children were invited to participate in this study. Children with any chronic disease and acute infection were excluded from the study. After obtaining written informed consents from their parents, fasting blood samples of participant children were drawn to measure serum leptin, ghrelin and calprotectin levels. Body mass indexes (BMI) of the children were calculated as weight (kg)/height^2^ (m). Skin prick test to aeroallergens and spirometry were performed to all participant children. Children were divided into two groups according to the responses to the question in ISAAC questionnaire “Has your child ever been diagnosed in asthma?” children were divided into two groups: group 1 included the children with previous diagnosis of asthma, while group 2 those children without previous diagnosis of asthma. The data of these two groups were compared.

### Laboratory procedures

All laboratory investigations were performed by a researcher unaware of the asthma diagnosis of the children. Five mL samples of peripheral venous blood samples were obtained from all children. Each blood sample was left to coagulate for 30 minutes, then centrifuged for 15 minutes at 10000 rpm and the extracted serum was collected and stored at −20°C until analysis.

Leptin (ng/mL) and ghrelin (ng/mL) were measured by EIA ( DRG Diagnostics, Inc., Marburg, Germany and Raybiotech, Inc., GA, USA respectively), whereas caprotectin (ng/mL) was measured by ELISA (Human Calprotectin ELISA kit, Cusabio Biotech, China) in serum samples. The methods of measurement were carried out according to the manufacturer instructions. The standard curves are created by reducing the date using computer software (Softmax Pro.) capable of generating four parameter logistic (4PL) curve-fit. The lower detection limits were 1 ng/mL for leptin, 161 pg/mL for ghrelin, and 2 ng/mL for calprotectin. The values under lower detection limits were accepted as 0 ng/mL.

### Skin prick test

Skin prick tests to aeroallergens were performed by a researcher unaware of the asthma diagnosis of the children with 24 common aeroallergens belonging to 5 groups; mites (*Dermatophagoides farinae, Dermatophagoides pteronyssinus*), molds (*Alternaria, Aspergillus mix, Penicillium mix, Candida albicans*), pollens (*Betulaceae, Aesculus Hippo, Olea Europea, Plantago, Artemisia, Parietaria, Secale cereale, Triticum vulgaris, Zea mays, mixture of 5 grasses*), animal epithelia (feathers mixture [duck, goose and hen], cat hair, dog hair) and insects (cockroach) (Stallergenes, Antony, France). Histamine and saline were used as positive and negative controls, respectively. A drop of each allergen extract was placed on the volar surface of the left forearm and was penetrated with a stallerpoint. After 15 minutes, the wheal reaction was measured as the mean of the longest diameter and the diameter perpendicular to it. A wheal diameter of at least 3 mm greater than those of the negative controls was considered as positive.

### Lung function testing

Lung function was assessed with a spirometer (Vmax Encore 22, California, USA). At least three reproducible blows with maximum difference between two best measurements of 5% or 150 ml were obtained and the highest value of these three attempts was accepted. Measurements of forced expiratory volume in one second (FEV_1)_, forced vital capacity (FVC), peak expiratory flow (PEF) and forced expiratory flow between 25 and 75% of vital capacity (FEF_25-75_) were compared to normal values, standardized for gender, height and age, and were expressed as a percentage of the predicted value.

### Statistical analysis

The Pearson chi-square test was performed in the case of categorical variables. Mean ± SD values were calculated for normally distributed data and median values for non-normally distributed data. Unpaired t-tests were used to test differences between groups for normally distributed variables. The Mann–Whitney U-test and Spearman’s rho test were used for non-parametric variables. p ≤ 0.05 was considered statistically significant. Statistical analyses were performed by using the SPSS software package for Windows (release 17.0.0; SPSS Inc., Chicago, Ill, USA).

## Results

One thousand and two hundred ISAAC questionnaires were distributed and 580 of them returned filled in. Parents of 74 children accepted to participate in the study following the invitation and the74 children (23 in Group 1 and 51 in Group 2) were recruited to the study. There was no statistical difference in serum levels of leptin, ghrelin, calprotectin, FEV_1_, FVC, PEF , FEF_25-75_ values , and skin prick test results between the two groups (p was 0.39, 0.72, 0.5, 0.17, 0.5, 0.27, 0.18, and 0.81 respectively). Data are presented in Table 1. Serum levels of leptin, ghrelin and calprotectin were also compared after dividing children into two new groups according to the results of skin prick tests: “Skin prick test positive” (SPT+) (n = 47) and “Skin prick test negative” (SPT-) (n = 24), and again there was no statistically significant difference between the two groups. Data are presented in Table 2.

**Table 1:** Comparison of BMI, gender, age, serum levels of leptin, ghrelin and calprotectin levels, lung function and skin prick tests in asthmatic and non-asthmatic children

	**Group 1 (n = 23)**	**Group 2 (n = 51)**	**p value**
**BMI (mean ± SD)**	20.3 ± 4.4	19.1 ± 4.2	0.82
**Gender (girl)(n)(%)**	9 (39%)	31 (60%)	0.08
**Age (year)(mean ± SD)**	8.2 ± 1.2	8.8 ± 1.4	0.09
**Leptin (ng/mL) (median [minimum-maximum])**	5.3 (0.4-27.4)	8.8 (0.3-31.3)	0.39
**Ghrelin (ng/mL) (median [minimum-maximum])**	30.9 (1.9-138.5)	26.9 (1.0-436.3)	0.72
**Calprotectin (ng/mL) (median [minimum-maximum])**	1.0 (0.0-106.8)	1.5 (0.0-27.5)	0.5
**FEV**_**1**_**% (median [minimum-maximum])**	107 (76–126)	101 (79–130)	0.17
**FVC% (median [minimum-maximum])**	99 (73–121)	97 (36–173)	0.5
**PEF% (median [minimum-maximum])**	99 (72–126)	96 (78–126)	0.27
**FEF**_**25-75**_**% (median [minimum-maximum])**	111 (39–177)	95 (65–129)	0.18
**Skin prick test positive (n) (%)**	32 (65%)	15 (68%)	0.81

*BMI* body mass index, *FEF*_*25-75*_ forced expiratory flow between 25 and 75% of vital capacity, *FEV*_*1*_ forced expiratory flow in one second, *FVC* forced vital capacity, *PEF* peak expiratory flow.

**Table 2:** Comparison of serum levels of leptin, ghrelin and calprotectin levels according to the skin prick test results

	**SPT + (n = 47)**	**SPT- (n = 24)**	**p value**
**Leptin (ng/mL) (median [minimum-maximum])**	5.2 (0.2-27.5)	5.7 (0.8-20.9)	0.92
**Ghrelin (ng/mL) (median [minimum-maximum])**	30.9 (1.9-138.5)	26.9 (1.0-436.3)	0.98
**Calprotectin (ng/mL) (median [minimum-maximum])**	1.0 (0.0-19.4)	0.5 (0.0-106.8)	0.62

SPT+, Skin prick test positive, SPT-, Skin prick test negative.

**Figure 1:**
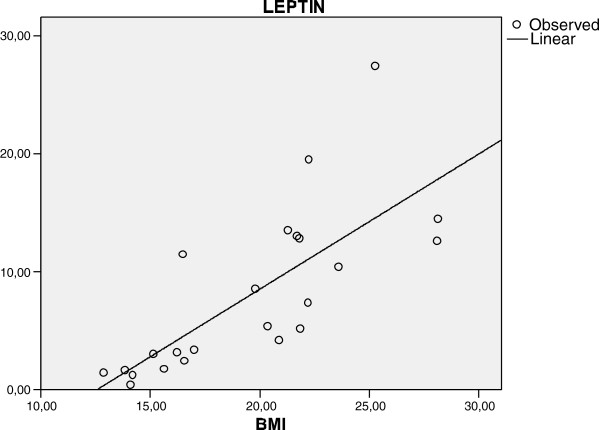
**Correlation between the serum levels of leptin and BMI in all participant children.** (p < 0.001 and correlation coefficient = 0.85).

There was a significant correlation between BMI and serum leptin levels (p < 0.001 and correlation coefficient = 0.85) (Figure 1) of all participants, but not between BMI and serum ghrelin and calprotectin levels (p values are 0.27 and 1.0, correlation coefficients are 0.12 and 0.00 respectively).

## Discussion

In this cross-sectional study, no differences in serum levels of leptin, ghrelin and calprotectin were detected between children with and without a previous diagnosis of asthma according to ISAAC questionnaire.

Previously, a growing number of studies have examined the potential role of leptin in the respiratory system. Although leptin is synthesized and secreted mainly by white adipose tissue [11], accumulative data have identified foetal and adult lung tissue as leptin responsive and producing organs. Also, leptin’s involvement in pulmonary homeostasis has become increasingly evident [12-15]. Leptin and leptin receptors are expressed by the lung [12,16]. Besides its proinflammatory systemic effects, leptin negatively modulates the function of regulatory T cells that are associated with asthma [17], and promote Th1 proliferation with increased production of interferon-γ [18,19]. Recent *in vitro* studies indicate that leptin also stimulates release of vascular endothelial growth factor by airway smooth muscle cells [20], although it does not affect airway smooth muscle cell proliferation [13]. Vascular endothelial growth factor may stimulate subepithelial neovascularization and vascular permeability, a key finding in asthma [20]. *In vitro* studies have documented that leptin can significantly up-regulate the cell surface expression of intracellular adhesion molecule ICAM-1 and CD18 and suppress those of ICAM-3 and L-selectin in eosinophils [21], while it augments alveolar macrophage leukotriene synthesis [22]. The latter results suggest that leptin may induce accumulation of eosinophils and may enhance inflammatory processes at sites such as the lung or the airways, thereby increasing to some extent allergic airway responses [21,22]. A causal role for leptin in asthma is supported by murine studies: the administration of exogenous leptin in leptin-deficient mice augments airway hyperreactivity following allergen challenge, as well as lung inflammation following ozone exposure [1,23]. Human data are, however, currently inconclusive regarding the independent association between serum leptin concentration and risk of asthma [3,24,25]. A large population-based study by Jartti et al. [26] used a sequential nested case control study design set within an established Finnish cohort and did not show an independent association between asthma and serum leptin concentrations. Some children studies with significantly smaller sample sizes showed a positive association [3,24], while others not [27]. In our cross-sectional study, we also did not show an correlation between asthma (both according to self declaration of doctor diagnosed asthma or to the results of skin prick tests) and serum leptin concentration.

Undoubtedly, leptin has emerged in the literature as a multifunctional hormone with versatile activities and complex counteractions with other cytokines and adipokines. However, decoding its pulmonary impact is not an easy task, since the role of leptin cannot always be separated from obesity and the biology of adipose tissue. Recent epidemiological studies have demonstrated that the prevalence of asthma and obesity are both increasing concomitantly, suggesting that these factors may be causally related [28]. Accumulating evidence has implicated systemic changes in immune function in the development of obesity; several obesity-related hormones and cytokines may lead to airway hyper-responsiveness [1,29,30]. In our study there was no difference between the BMIs of asthmatic and non-asthmatic children, so we could eliminate the effect of obesity on the serum levels of leptin.

In the present study, we paid special attention to the recently discovered hormones ghrelin and leptin, because both hormones seem to exert a wide range of effects on the immune system [4,31]. It has been reported that ghrelin dose-dependently inhibits proliferation of anti-CD3-activated murine T cells and non-specifically inhibits both T-helper 1 (Th1; IL-1 and IFN-γ) and Th2 (IL-4 and IL-19) cytokine mRNA expression [32]. In a previous study on children, Matsuda et al. [2] showed a significant correlation between the levels of plasma ghrelin and leptin levels and BMI. They found that overweight children had significantly higher IgE concentrations than non-obese children, and also observed significant correlation between serum IgE and plasma concentrations of ghrelin and leptin. These findings suggested the possibility that obesity-related hormones may represent a link between obesity and allergic disorders. Nevertheless, Okamatsu et al. [33] found that serum leptin had a significant positive correlation with BMI while plasma ghrelin did not correlate with it. Surprisingly, a significant inverse correlation between plasma concentrations of ghrelin and serum immunoglobulin concentrations was found in their study. In the present study, we did not find a difference between the serum ghrelin levels of asthmatic and non-asthmatic children, and there was no correlation between serum ghrelin levels and BMI in the whole group.

Elevated serum calprotectin (synonyms in the literature: aka MRP8/14, calgranulin, cystic fibrosis-associated antigen, and S100) levels are found in a variety of chronic inflammatory conditions, including rheumatoid arthritis, allograft rejections, inflammatory bowel and lung diseases, and environmental tobacco smoke-exposure [34]. Asthma is a chronic inflammatory disease, therefore we compared the serum calprotectin levels of asthmatic and non-asthmatic children demonstrating no significant difference between the two groups. Possibly, we could not detect any difference between groups due to the low number of children with asthma. In addition, skin prick test positivity was equal in both groups, and this might be another reason why no differences were detected between two groups. It could be better to include children with current diagnosis of asthma rather than responses to questionnaires.

There were no statistically significant differences in terms of spirometric data and skin prick test results between the two groups. Because our study was based on a screening questionnaire only, it could be that, absence of current asthma and therefore allergic inflammation in some of the children at the time of study might have influenced the results. Possibly, monitoring inflammatory markers during exacerbations of asthma could be more useful.

Although we could not find any statistical difference between the two groups in terms of serum levels of leptin, there was a significant correlation between BMI and serum leptin levels. As leptin is synthesized and secreted mainly by white adipose tissue this correlation was rational and compatible with previous studies.

## Conclusions

It is indisputable that asthma is an inflammatory condition although we could not show the inflammation by using serum leptin, ghrelin and calprotectin levels in asthmatic children. According to the current literature, this is the first study aimed to show the association of serum calprotectin with asthma. As our study was a cross-sectional study, further prospectively designed randomized controlled studies are necessary in order to show the association between these markers and inflammation in asthma.

## Competing interests

The authors declared that they have no competing interests.

## Authors’ contributions

NC participated in the design of the study, performed statistical analysis and drafted the manuscript. NG and CD participated in coordination and helped to draft the manuscript. NNB participated in the design of the study and helped to draft the manuscript.
